# 

**Published:** 1894-01

**Authors:** 


### CONTENTS.

ORIGINAL ARTICLES—

Circumscribed Traumatic Aneurism of the Left Femoral Artery—Ligation of the External Iliac Artery—Gangrene of Leg, and Death on the 40th Day. By Howard J. Williams, M. D., Macon, Ga .......... 1
Two Recent Cases of Excision of the Vermiform Appendix for Chronic Relapsing Appendicitis in the Intervals. By Thomas G. Morton, M. D., Philadelphia, Pa .......... 4
Some Nervous Effects of Digestive Disorders. By A. K. Bond, M. D .......... 8
Carbolic Acid Used in Full Strength in Surgery. By O. H. Allis, M. D., Philadelphia, Pa .......... 11

SOCIETY REPORTS—

Philadelphia Academy of Surgery .......... 14–20
Clinical Society of Maryland .......... 21–23
Gynecological and Obstetrical Society of Baltimore .......... 23–26

EDITORIALS—

Open Letter upon the Prevention of Yellow Fever by Inoculation .......... 40
Higher Medical Education .......... 44
Eleventh International Medical Congress .......... 44
Association Georgia Central Railroad Surgeons .......... 45
Medical Association of Georgia .......... 47
New Advertisements .......... 48
To Subscribers .......... 48
Editorial Notes .......... 46–48

BOOK REVIEWS—

A System Genito-Urinary Diseases, Syphilology and Dermatology. Edited by P. A. Morrow, M. D. .... 27
The Pharmacopocia of the United States of America.... 28
Annual of the Universal Medical Sciences.... 28
New Truths in Ophthalmology. By G. C. Savage, M. D.... 29
Leonard's Physician Pocket Day-Book.... 29
The Medical News Visiting List for 1894.... 30

SELECTIONS AND ABSTRACTS—

How to find the Upper Extremities of the Divided Tendons in Cases of Division of the Flexor Tendon of the Fingers.... 26
Sulphate of Quinine in the Local Treatment of Ulcers.... 34
Funtional Dyspepsia, So-called.... 31
The Feeding of Infants.... 36
Things Worth Remembering.... 37
Extract From a Lecture on "Degeneration in Obesity.".... 36
Heredity of Syphilis.... 38
Paralysis Following Measles.... 38
A New Process for Measuring the Solubility of Salicylic Acid. 39
Undeveloped Mammæ.... 54
Recurring Grippe.... 56

PRESCRIPTION DEPARTMENT.... 50-51

For Sub-Acute Rheumatism—Infantile Convulsions—Local Anæsthesia of the Bladder—Diarrhœa in Children—For Headaehe—Treatment of Colds—As a General Tonic in Euelometritis—La Grippe—Acute Pleurisy—Acute Catarrh.

SPECIAL NOTES.... 52-56

